# Proposed Meeting of the American Medical Association

**Published:** 1863-03

**Authors:** 


### PROPOSED MEETING OF THE AMERICAN MEDICAL ASSOCIATION.

We received too late for insertion in the last number, and after the notice which it contained had been written, a call signed by Dr. N. S. Davis, and purporting to be authorized by the "Committee of Arrangements," for a meeting of the American Medical Association, to take place in Chicago on the first Tuesday in June, 1863. On enquiry of some members of said Committee we find they know nothing about said call. The Committee without any authority adjourned the meeting of 1861–1862; and now some of them, equally without authority, have announced a meeting under circumstances quite as unfavorable as have existed in former years.

If a meeting is desired we conceive it devolved upon the State societies, other organized bodies, including teachers in Colleges and Hospitals, to intimate their wishes to that effect. This, as far as we are informed, has not been done in more than one instance.

The Association meeting in June can have no claim to be considered "National," and must if convened but favor the effect of forever perpetuating its sectional character. For ourselves we do not despair of the return of better days, when our brethren now in the army and numerous Union men in the Southern States shall join with their brethren in the North in meetings truly National in their character. We know that some most active in the matter do not share this hope, probably do not desire such a result, but it is a conviction to which we shall cling to the last.

The excitement of civil war is not favorable to the calmness required in scientific meetings. The National Association of Science has thought it wise to suspend its annual assemblage. Several of the State societies have followed its example. From such no representatives can be expected.

The time appointed is peculiarly unfortunate, being the day on which a great "Canal Convention" is to convene in this city. Citizens will be called upon to open their houses for the accommodation of the members of this latter body. Several eminent medical gentlemen will find it necessary to devote most of their time to it. Many will desire to be present to listen to its proceedings. If, therefore, a general attendance is desired, we would suggest that the second Tuesday of June be substituted for the first Tuesday. We make this suggestion in the interest of the profession of the city who will desire to give to their friends a cordial and hospitable reception whenever they may do us the honor of a visit.

